# What we see is what we touch? Sex estimation on the skull in virtual anthropology

**DOI:** 10.1007/s00414-024-03244-w

**Published:** 2024-04-30

**Authors:** Sandra Braun, Nicole Schwendener, Fabian Kanz, Sandra Lösch, Marco Milella

**Affiliations:** 1 Department of Physical Anthropology, Institute of Forensic Medicine, University of Bern, Murtenstrasse 24-28, 3008 Bern, Switzerland; 2 Department of Forensic Medicine and Imaging, Institute of Forensic Medicine, University of Bern, Bern, Switzerland; 3 Forensic Anthropology Unit, Center for Forensic Medicine, Medical University of Vienna, Vienna, Austria

**Keywords:** Computed tomography, 3D surface scan, Morphoscopic sex estimation, Cranium, Tactility

## Abstract

**Background:**

The increased use of virtual bone images in forensic anthropology requires a comprehensive study on the observational errors between dry bones and CT reconstructions. Here, we focus on the consistency of nonmetric sex estimation traits on the human skull.

**Materials and methods:**

We scored nine nonmetric traits on dry crania and mandibles (*n* = 223) of archaeological origin and their CT reconstructions. Additionally, we 3D surface scanned a subsample (*n* = 50) and repeated our observations. Due to the intricate anatomy of the mental eminence, we split it into two separate traits: the bilateral mental tubercles and the midsagittal mental protuberance. We provide illustrations and descriptions for both these traits.

**Results:**

We obtained supreme consistency values between the CT and 3D surface modalities. The most consistent cranial traits were the glabella and the supraorbital margin, followed by the nuchal crest, zygomatic extension, mental tubercles, mental protuberance, mental eminence, mastoid process and ramus flexure, in descending order. The mental tubercles show higher consistency scores than the mental eminence and the mental protuberance.

**Discussion:**

The increased interchangeability of the virtual modalities with each other as compared to the dry bone modality could be due to the lack of tactility on both the CT and surface scans. Moreover, tactility appears less essential with experience than a precise trait description. Future studies could revolve around the most consistent cranial traits, combining them with pelvic traits from a previous study, to test for accuracy.

## Introduction

### Sex estimation

In 1970 [1], the occipital protuberance, mastoid process, glabella, supraorbital margin and mental eminence were described among other traits on the human skull for sex estimation. These five traits scored on a scale from 0 to 5 had originally been published by Broca [2]. Acsádi and Nemeskéri (1970) changed the scale from + 2 (hypermasculine) to -2 (hyperfeminine). The five traits were republished by Buikstra and Ubelaker [3], provided with a line drawing and reorganized on a scale of 1 (female) to 5 (male). Score 2 referred to probable female, 3 to ambiguous sex, and 4 to probable male [3]. At the same time, the occipital protuberance was renamed nuchal crest [3]. In 2008, Walker [4] combined the traits further into a method, included different population groups, reworded the trait descriptions slightly and applied statistical tests to quantify the resulting accuracy. Finally, in MorphoPASSE [5] the repeatability of observations was increased by the inclusion of photographic depiction of each trait together with their description. In addition, explicit descriptions of the intermediate scores 2, 3 and 4 were supplied [5].

### Imaging techniques in anthropology

The application of imaging techniques within forensic anthropology has become prevalent since the turn of the millennium [6–14], favored by advantages such as global data accessibility [15] and the non-invasive nature of imaging techniques [16]. While identified osteological collections have traditionally been used for forensic anthropological research [17], they do not always reflect a present-day context, thus potentially distorting the applicability of research output for modern forensic circumstances [18]. Relating to the increasing ethical concern revolving around identified osteological collections and human remains in general [19, 20], more virtual collections, mostly consisting of computed tomography (CT) scans, have been established in recent years [21–24]. In parallel, open-source software packages have become available, allowing the analysis of CT scans for forensic anthropological research [21] and the application of sophisticated morphometric protocols [25]. This ongoing trend requires a thorough investigation in the comparability of commonly used methods to estimate the biological profile between the analogous (dry bone) and the virtual modalities. However, a wider ethical consensus regarding data sharing and 3D printing has yet to be agreed upon [24], and data safety and storage must be warranted perpetually [26]. In addition, the lack of tactility on virtual bone reconstructions may influence our perception of a feature [27–30]. For instance, dry bones or 3D prints of bone models are more suitable educational material for osteology students than 3D models on a screen [29]. However, the influence of tactility for advanced osteologists as they use virtual bone models for their research is largely unknown and constitutes the target of our study. It is therefore essential to assess the errors associated with methods developed on dry bones when applied to virtual modalities. In this paper, we analyze cranial sex estimation methods to virtual modalities and investigate the interchangeability of modalities [27, 31, 32].

Earlier studies have tested the efficacy of cranial sex estimation methods applied to a virtual environment [11, 33–35] without, however, repeating observations on dry bone for direct comparison. Thus, these studies did not focus on the interchangeability of modalities. Other studies have considered modality interchangeability (e.g. the similarity of observations across modalities), comparing dry bones with virtual images, but have used relatively small sample sizes [9, 29, 36–38]. To the best of our knowledge, only one study so far was dedicated to the comparison of the dry bone and micro-focus X-ray computed tomography (micro-XCT) on a larger sample (N = 105), although limiting the focus to the mental eminence [27]. Their results suggested a low consistency for the scoring of the mental eminence across the two modalities [27]. Considering this finding, we seek an amelioration of the trait and attempt to divide the traditional mental eminence trait into the bilateral mental tubercles and the midsagittal mental protuberance. We do this in an attempt to improve the consistency of this trait across the modalities.

It is worth stressing that our focus is *not* the evaluation of possible differences between scoring protocols in their performance of accurately discriminating between sexes. Rather, our concern is establishing which type of error (within and among observer, and between modalities) affects the evaluation of each feature by using, for the first time, an extensive dataset. Thus, the aim of this work is the exploration of the presence and type of deviations in the scoring of sexually dimorphic traits on the cranium and mandible when observed on the analogous (dry bone) and virtual (CT) modalities. As an additional pilot comparison, we added a subsample of 3D surface scans to the study to have an idea of how virtual modalities compare with each other. In particular, this study builds up around three research questions:

- What is the error when observing the sex estimation traits on skulls and on CT reconstructions of the same specimens, e.g., are these two modalities interchangeable for the scoring protocols under analysis?
- As an additional pilot project on a subsample, what is the error when observing the same scoring protocols to 3D surface scans, as compared to dry bones and CT reconstructions?
- Can we score the mental tubercles and the mental protuberance on the mandible more consistently on the different modalities (dry bone, CT and surface scans) than the traditional mental eminence trait, e.g., are the modalities interchangeable for the two separate traits?

## Materials and methods

### Materials

The forensic database of the Institute of Forensic Medicine (IRM) in Bern consists of postmortem CT (PMCT) datasets and forensic reports; no macerated dry bones are available for analysis. The latter are, however, a prerequisite for a comparison between observation modalities. Considering this issue, and the fact that our focus is *not* the estimation of sex (which would require an identified sample), but the *quantification of the error* affecting the scoring of features routinely used to estimate sex, we decided to base our study on a large osteoarchaeological sample. This includes 223 paired crania and mandibles from archaeological burial sites in Switzerland, dating between the seventh and the nineteenth centuries CE (Table 1). For each context, estimates of demographic parameters (age-at-death and sex) are available from previous anthropological reports [39–41]. Individuals were included in this study based on their relatively good preservation and estimated age-at-death of ca. 18 years and older. We excluded specimens exhibiting pathologic features possibly affecting the cranial and/or mandibular morphology (e.g., fractures, metabolic conditions, or developmental anomalies).

**Table 1:** Archaeological sites from which the specimens in our study originate, including chronologies, number of female and male specimen, and modalities (dry bone, CT and surface scans)

Site	Period	F	M	Dry bone	CT	Artec 3D
Bern Grosse Schanze	18th-19th cent	6	13	x	x	
Biel-Mett Kirche	7th-9th/13th-14th cent	15	14	x	x	
Büren Chilchmatt	8th-16th cent	8	13	x	x	
**Ins Kirchgemeindehaus**	**6th-10th cent**	**23**	**27**	**x**	**x**	**x**
Kallnach Bergweg 95	6th-10th cent	17	20	x	x	
Köniz Kirche	6th-14th cent	11	0	x	x	
Miscellaneous		2	0	x	x	
Nidau	16th-17th cent	3	0	x	x	
Steffisburg	7th-11th cent	8	0	x	x	
Twann St. Petersinsel	8th-14th cent	9	33	x	x	
Zweisimmen	8th-16th cent	1	0	x	x	

Bold: site used for intra- and interobserver, as well as intermodality agreements on all three modalities.

### Methods

#### CT and 3D surface scanning

The crania and mandibles were CT scanned separately, with a Somatom Definition AS 64 (Siemens, Berlin/Munich, Germany) with the following parameters: 140 kV, 118–216 mAs, slice thickness: 0.6 mm, increment: 0.3 mm; 512 × 512 pixel matrix, field of view 200 mm to 400 mm. We exported all raw data from PACS IDS 7 v. 20.2.8.3353 (Sectra, Linköping, Sweden), reconstructing them in Avizo (Thermo Fisher Scientific Inc., Waltham, Massachusetts, USA). Additionally, for a subset of 50 crania and mandibles (23 female, 27 male), we performed surface scans (Table 1), using an Artec Space Spider scanner (Artec 3D, Luxembourg) with a setting of eight frames per second. We reconstructed the scans with Artec Studio 15 software (Artec 3D, Luxembourg). For the scoring of all the 3D models (both CT and surface scans), we used the Artec Studio software.

#### Scoring protocols

For each specimen, we scored cranial and mandibular traits based on the protocols of Loth and Henneberg [42], Walker [4, 5] and Langley et al. [43]:

The method by Loth and Henneberg [42] attempts to quantify the degree of sexual dimorphism of the mandibular ramus, which is scored based on its relative flexure with respect to the occlusal plane. Accordingly, the left and right ramus can be "flexed" (+ 1) or "straight" (-1), the two scores corresponding to male and female, respectively. Given the known effect of intra vitam tooth loss on mandibular morphology [44–46], we scored the ramus flexure only for individuals featuring an Eichner Index [47, 48] of A1 (no intra vitam loss of premolars or molars) and A2 (a maximum of one antagonistic contact in the premolars and molars lost intra vitam).

**Fig. 1:**
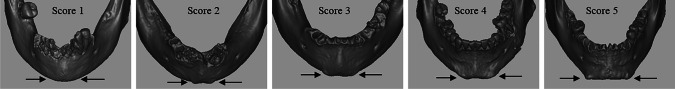
CT scans of mental tubercles (black arrows) scores 1 to 5 with increasing expression of the trait, independent from mental protuberance. Individuals Ins Kirchgemeindehaus (3465, 3466, 3469) and Twann (3365 and 3371), respectively

**Fig. 2:**
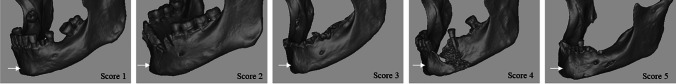
CT scans of mental protuberance (white arrows) scores 1 to 5 with increasing expression of the trait, independent from mental tubercles. Individuals Ins Kirchgemeindehaus (3543, 3472, 3469, 3529) and Steffisburg (3975), respectively

The method by Walker (2008) encompasses five traits (nuchal crest, mastoid process, supra-orbital margin, glabella, and mental eminence), scored according to an ordinal scale from 1 (female/gracile) to 5 (male/robust), with scores 2 and 4 corresponding to "probably female" and "probably male" morphologies, respectively, and score 3 to "indeterminate". We based our scoring on the criteria listed by Walker (2008) and MorphoPASSE [5]. Considering the finding by Braun et al. (2022), we will divide the mental eminence trait into the mental tubercles and the sagittal mental protuberance in our study, in addition to scoring the traditional mental eminence. With this attempt, we keep two features apart that make the human menton *'much more complex'* in its expression [49] than the description in Walker [4] might suggest. To score the expression of the mental tubercles and the mental protuberance, we applied the same scoring protocol (scores 1 to 5), with minimal and maximal expression of the traits corresponding to score 1 and score 5, respectively (Figs. 1 and 2, Table 2).

**Table 2:** Description for the mental tubercles and mental protuberance

Score	Mental tubercles	Mental protuberance
1	No tubercles detectable visually and tactilely; chin is rounded	No sagittal protuberance detectable; the area between and above the tubercles is not elevated
2	Small tubercles are detectable visually and tactilely	Slight sagittal protuberance in the area between and above the tubercles
3	Intermediate sized tubercles well distinguishable visually and tactilely	Intermediate sized sagittal protuberance, well detectable visually and tactilely
4	Large tubercles, hinting at a squaring of the menton, detectable visually	Large sagittal protuberance, bulging detectable visually
5	Extreme tubercles, delineating a square menton, detectable visually	Extreme sagittal protuberance, detectable visually

We scored the zygomatic extension according to Langley and colleagues [43]. The ordinal scoring scale corresponds to score 1: "an absent ridge or extension" and score 5: a "robust and prominent ridge" [43], with scores 2, 3 and 4 described in detail.

Whenever available, we scored the mastoid process and supraorbital margin on the left side, using the right otherwise. For the zygomatic extension, we scored the right side [43]. We scored the bilateral mental tubercles and ramus flexure on both sides.

### Data analysis

#### Intra- and interobserver agreement

We quantified the intra- and interobserver agreements based on a subsample of 50 skulls. Two observers carried out the observations independently on the dry bone and the virtual modalities. A first observer (SB) scored these 50 specimens twice per modality (Artec 3D surface scans [A], dry bone [B] and CT [C]), at an interval of at least two weeks between observations. A second observer (MM) scored the 50 individuals once per modality (Table 3).

**Table 3:** Intra- and interobserver, as well as intermodality agreement tests

Agreement test	Modalities	Abbrev	*N* skulls
Intraobserver	Artec 3D—Artec 3D	AA	50
Intraobserver	Dry bone—dry bone	BB	50
Intraobserver	CT—CT	CC	50
Interobserver	Artec 3D—Artec 3D	AA*	50
Interobserver	Dry bone—dry bone	BB*	50
Interobserver	CT—CT	CC*	50
Intermodality	Artec 3D—dry bone	AB	50
Intermodality	Artec 3D—CT	AC	50
Intermodality	Dry bone—CT	BC	223

#### Intermodality agreement

**Fig. 3:**
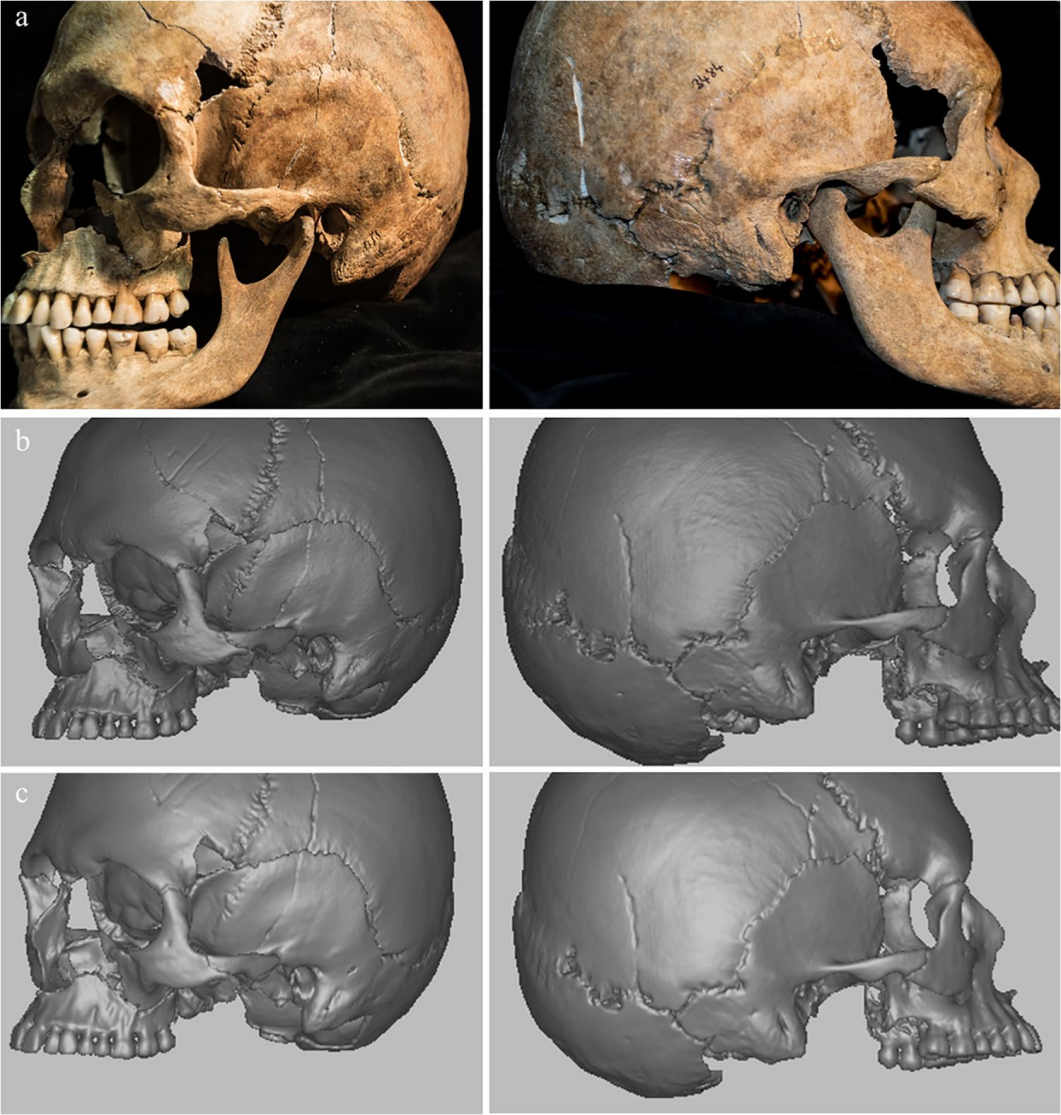
Cranium of specimen Ins Kirchgemeindehaus (3484) as dry bone (**a**), CT scan (**b**) and 3D surface scan (**c**)

We assessed the agreement between the observations on dry bone, CT and surface scan models by comparing the scores assigned to the same specimens on each modality (Fig. 3). For this purpose, we used the data collected by the first observer during the first scoring. Since surface scans are available only for a subset of 50 individuals, the sample size for each comparison differs (Table 3). The intermodality agreement was tested between 3D surface scans and dry bone (abbreviation: AB, n = 50), 3D surface and CT scans (AC, n = 50) and dry bone and CT scans (BC, n = 223).

We applied Cohen's kappa *κ* [50] tests to calculate the agreements in the scoring of categorical variables [42]. For the traits in Walker [4] and Langley et al. [43], which are ordinally scored, we applied Cohen's weighted *κ* [50] tests. We set a threshold value for acceptable agreement at *κ* ≥ 0.6 [30, 51, 52], translating into substantial to almost perfect agreement according to Landis and Koch [53]. We compared out observations with the dry bone modality, which we considered the baseline because the protocols were developed on that environment.

#### Trait performance

We analyzed the individual traits and their *κ*-values across all tests in order to analyze the error associated with the comparisons across observers and between the analogous and the virtual modalities.

#### Consistency and availability

For the trait consistency, we scored a 1 for *κ*-values greater than 0.6, and a 2 for *κ*-values below 0.6. Moreover, we also investigated how often a trait was available for observation and combined this analysis with that of trait consistency. If a trait is not readily observable due to fragmentation, its value is questionable even if it exhibits a high degree of consistency. Consequently, we classified the traits into three groups: 1 (*'mostly available'*: availability > 80%), 2 (*'fairly often available'*: availability between 60 and 80%), and 3 (*'not readily available'*: availability < 60%). Thus, traits could vary between a minimum score of 2 for great consistency and availability, and a maximum score of 5 for poor consistency and availability.

For all analyses and figures we used the packages *irr* [54] for the agreement analyses and *fmsb* [55] and *ggplot2* [56] for the graphics in R (version 4.1.4)

## Results

### Intraobserver agreements

All mean *κ*-values for the left and right mandibular ramus flexure were below the acceptable threshold of 0.6 (mean 0.479, standard deviation [SD] 0.131, mean 2.53, SD 0.020 and mean 0.409, SD 0.198). For the 3D surface scan and the CT tests, we obtained *κ*-values greater than 0.6 (mean 0.702, SD 0.124 for AA and mean 0.642, SD 0.108 for CC). The dry bone comparison (BB) was below 0.6 (mean 0.415, SD 0.188).

### Interobserver agreements

In the interobserver agreement tests (AA*, BB*, CC*), the mean *κ*-values for all three comparisons of ramus flexure and ordinal traits were lower than 0.6.

### Intermodality agreements

The negative *κ*-value (-0.087) in the 3D surface-CT scan (AC) comparison suggests an agreement lower than chance [53]. The highest mean values for the intermodality tests were 0.667 for the categorical and 0.643 for the ordinal traits. The remaining comparisons yielded mean* κ*-values below 0.6.

### Analysis per trait

**Fig. 4:**
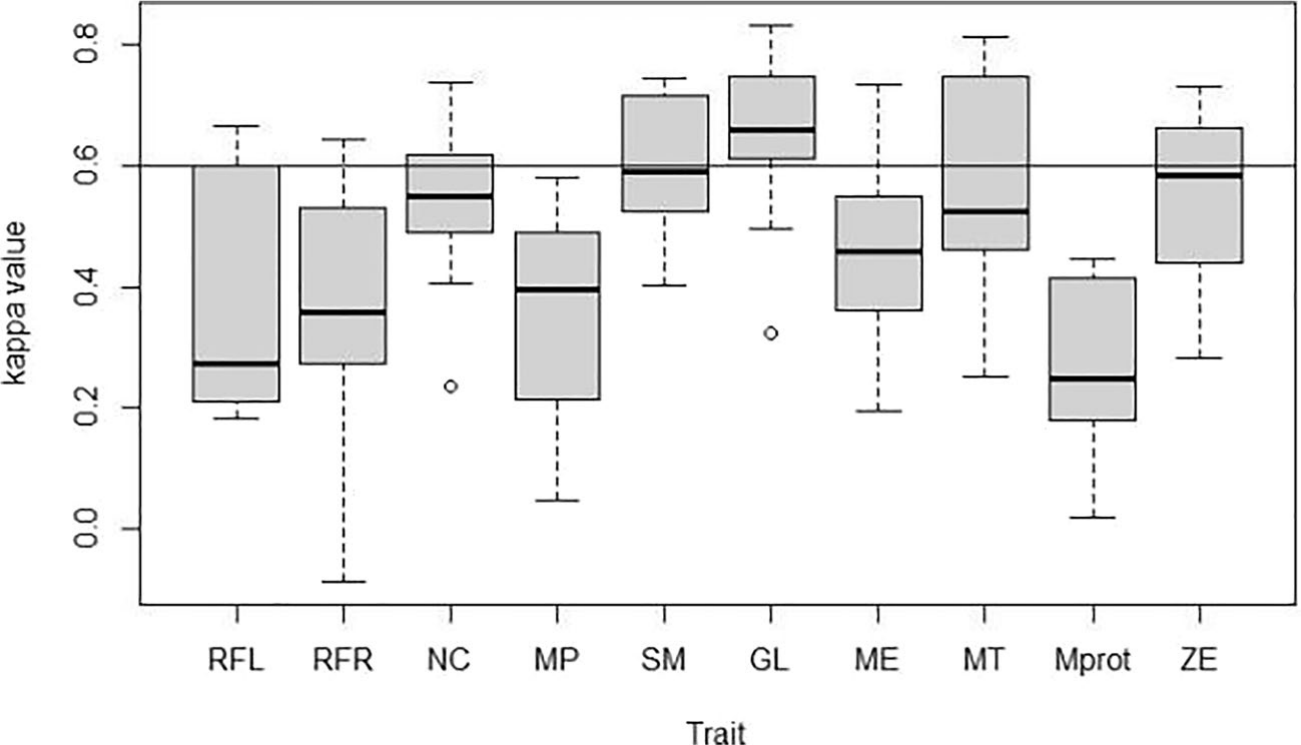
Trait performance for mandibular ramus flexure left and right (RFL/RFR) and ordinal traits of the cranium. Horizontal line indicates *κ*-value 0.6. RFL = ramus flexure left; RFR = ramus flexure right; NC = nuchal crest; MP = mastoid process; SM = supraorbital margin; GL = glabella; ME = mental eminence; MT = mental tubercles; Mprot = mental protuberance; ZE = zygomatic extension

The analysis per trait (Fig. 4) shows that only the glabella and the supraorbital margin yield a mean *κ*-value above the acceptable threshold of 0.6 (mean 0.643, SD 0.138, and mean 0.604, SD 0.105, respectively). All other traits are below this value. We find that the mental tubercles fare better (mean 0.553, SD 0.170) than the mental eminence (mean 0.473, SD 0.149), while the mental protuberance (mean 0.258, SD 0.137) results in an even lower agreement.

**Fig. 5:**
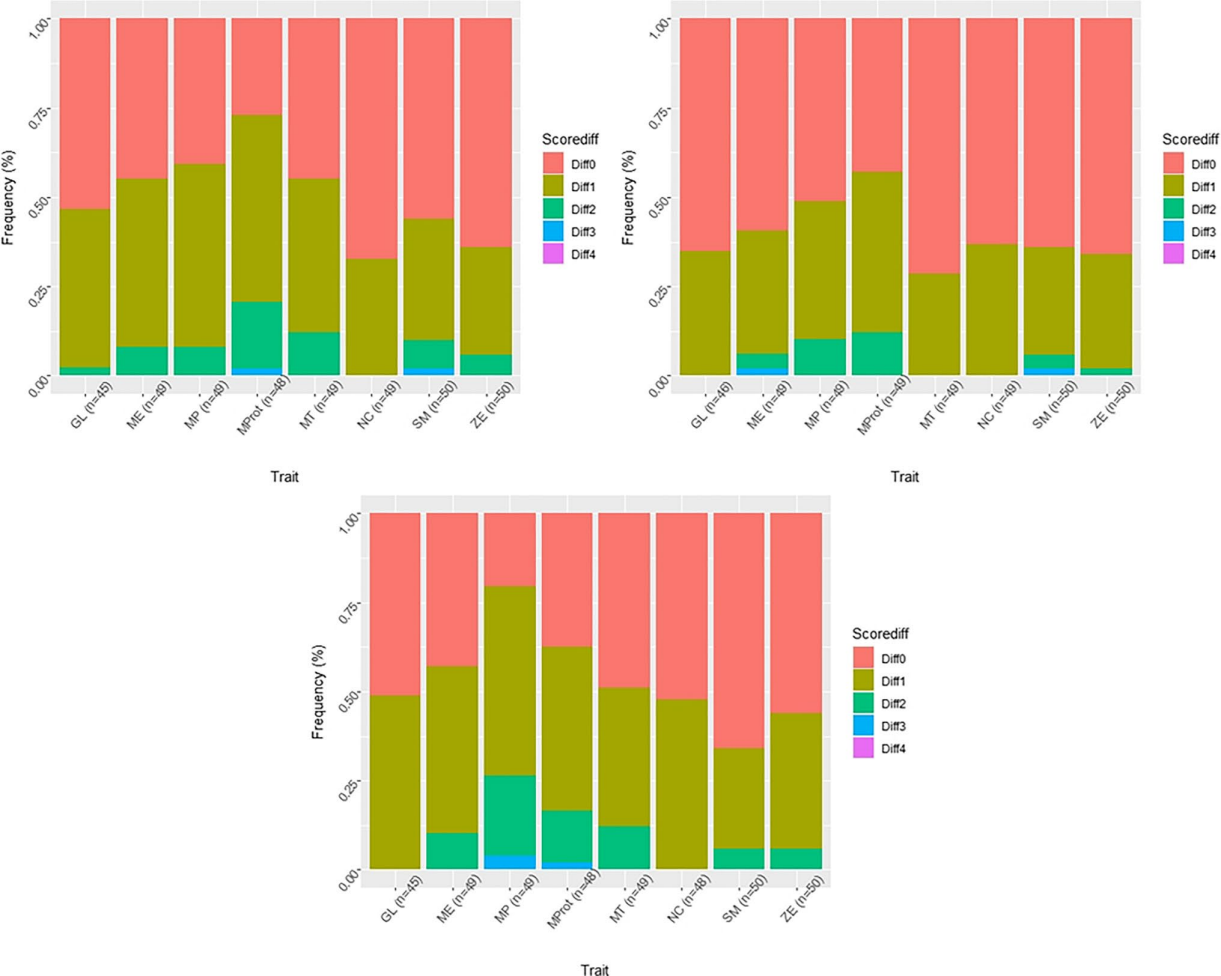
Plots of the ordinally scored traits of the cranium and the mandible. Comparison top left: surface scan-dry bone (AB); top right: surface scan-CT (AC); bottom: dry bone-CT (BC). Diff0 indicates no difference in scoring, Diff4 the maximum difference in scoring between two scorings (e.g. score 1 and score 5 or vice versa). GL = glabella; ME = mental eminence; MP = mastoid process; Mprot = mental protuberance; MT = mental tubercles; NC = nuchal crest; SM = supraorbital margin; ZE = zygomatic extension.

Figure 5 shows the trait performance per modality comparison, highlighting the relative frequency of the differences between scores, with the highest proportion of zero difference between scores (Diff0), and the maximal difference of 4 scores (Diff4) between modalities.

This comparison clarifies that 3D surface and the CT scans yielded the highest frequency of Diff0 and a lower frequency of Diff2 and Diff3. In none of the instances did we assign maximally different scores in our observation (Diff4).

In order to further explore the differences in trait performance, we evaluated the *κ*-values established in our study (Table 4). The glabella had the highest number of *κ*-values greater than 0.6, while the mastoid process and the mental protuberance performed below the acceptable agreement threshold across all tests. The results for the supraorbital margin and the zygomatic extension were intermediate. Moreover, the mental tubercles performed better than the mental eminence.

**Table 4:** *κ*-values per trait in descending order per trait; *n* refers to trait availability for BC comparison (*n* = 223), in bold. *Italic font* indicates *κ*-values < 0.6

RFL		RFR		NC		MP		SM		GL		ME		MT		Mprot		ZE	
Mod	(*n* = 54)	Mod	(*n* = 58)	Mod	(*n* = 213)	Mod	(*n* = 215)	Mod	(*n* = 217)	Mod	(*n* = 209)	Mod	(*n* = 218)	Mod	(*n* = 217)	Mod	(*n* = 217)	Mod	(*n* = 221)
**BC**	**0.667**	**BC**	**0.643**	AA	0.738	*AA*	*0.583*	CC	0.745	AA	0.833	AA	0.737	AA	0.815	*CC*	*0.445*	AA	0.732
AA	0.609	CC	0.607	AB	0.680	*CC*	*0.512*	AA	0.744	CC	0.754	CC	0.666	AC	0.776	*AA*	*0.431*	CC	0.667
AA*	0.600	BB*	*0.530*	AC	0.619	*AC*	*0.490*	**BC**	**0.718**	BB*	0.749	*AC*	*0.549*	CC	0.749	*BB*	*0.415*	BB	0.664
BB*	*0.273*	CC*	*0.530*	*CC*	*0.596*	BB*	*0.474*	AC	0.641	AC	0.708	***BC***	***0.521***	***BC***	***0.562***	*AC*	*0.312*	AC	0.654
CC*	*0.273*	AA*	*0.357*	BB*	*0.558*	*AB*	*0.441*	AB	0.609	**BC**	**0.694**	*AB*	*0.482*	*AB*	*0.506*	***BC***	***0.252***	AB	0.611
*BB*	*0.233*	*AA*	*0.348*	***BC***	***0.544***	AA*	*0.353*	BB*	*0.569*	AB	0.627	AA*	*0.432*	AA*	*0.471*	AA*	*0.181*	***BC***	***0.560***
*CC*	*0.211*	*BB*	*0.273*	CC*	*0.539*	***BC***	***0.221***	AA*	*0.525*	BB	0.612	*BB*	*0.361*	*BB*	*0.462*	*AB*	*0.178*	BB*	*0.439*
*AC*	*0.182*	*AB*	*0.200*	AA*	*0.406*	CC*	*0.214*	*BB*	*0.518*	AA*	*0.497*	BB*	*0.356*	BB*	*0.395*	BB*	*0.096*	CC*	*0.290*
*AB*	*0.182*	*AC*	*-0.087*	*BB*	*0.236*	*BB*	*0.048*	CC*	*0.402*	CC*	*0.324*	CC*	*0.195*	CC*	*0.252*	CC*	*0.019*	AA*	*0.282*

RFL = ramus flexure left; RFR = ramus flexure right; NC = nuchal crest; MP = mastoid process; SM = supraorbital margin; GL = glabella; ME = mental eminence; MT = mental tubercles; Mprot = mental protuberance; ZE = zygomatic extension. AA = intraobserver agreement on the 3D surface scan modality; BB = intraobserver agreement on the dry bone modality; CC = intraobserver agreement on the CT modality. AA* = interobserver agreement on the 3D surface scan modality; BB* = interobserver agreement on the dry bone modality; CC* = interobserver agreement on the CT modality. AB = intermodality agreement on the 3D surface scan and dry bone modalities; AC = intermodality agreement on the 3D surface and CT scan modalities; BC = intermodality agreement on the dry bone and CT scan modalities.

### Consistency and availability

**Fig. 6:**
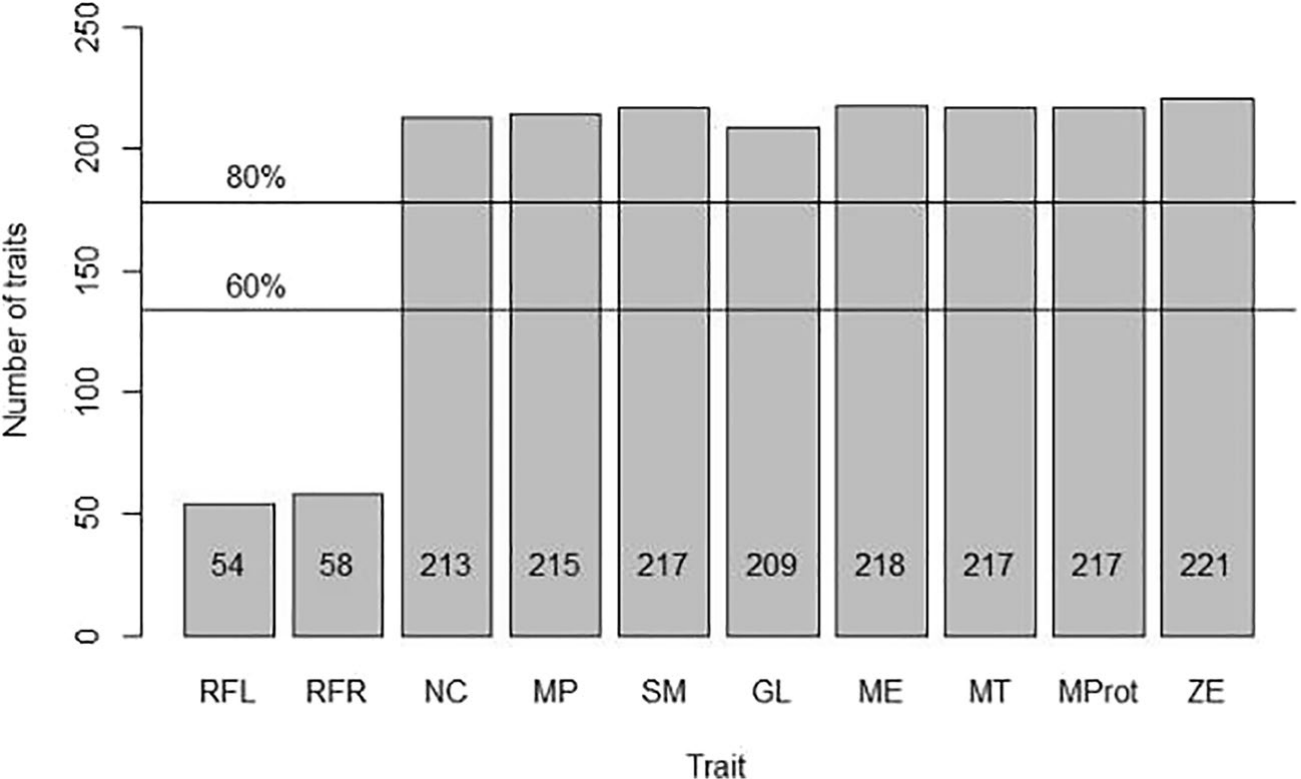
Trait availability of the categorical (RFL, RFR) and the ordinal traits on dry bone. Horizontal lines indicate 60% and 80% trait availability. RFL = ramus flexure left; RFR = ramus flexure right; NC = nuchal crest; MP = mastoid process; SM = supraorbital margin; GL = glabella; ME = mental eminence; MT = mental tubercles; Mprot = mental protuberance; ZE = zygomatic extension

The ramus flexure left and right were available in 54 and 58 of the 223 individuals (24.2% and 26.0%, respectively). In contrast, the nuchal crest, mastoid process, supraorbital margin, glabella, mental eminence, mental tubercles, mental protuberance and zygomatic extension were available in over 80% of cases (Fig. 6).

**Fig. 7:**
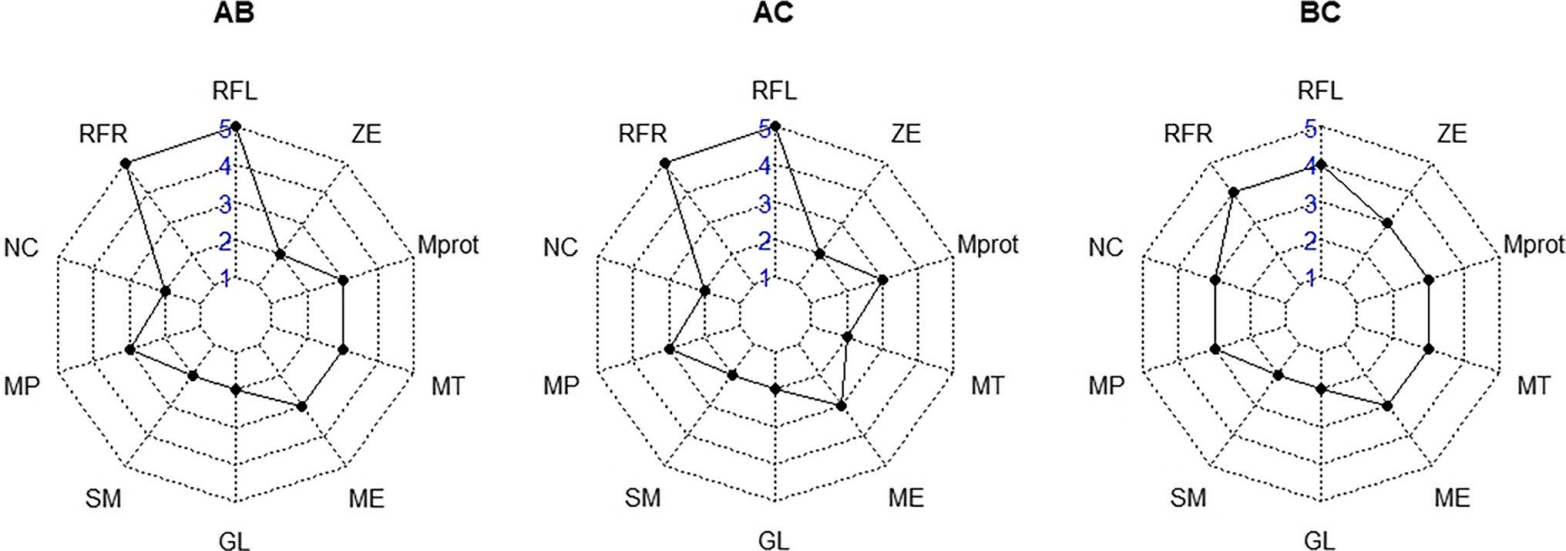
Spiderwebs of trait consistency and availability in the three comparisons (AB = surface scans-dry bone; AC = surface-CT scans; BC = dry bone-CT scans). Scores range from 2 (highest) to 5 (poorest). GL and SM performed relatively well throughout; RF poorest. RFL = ramus flexure left; RFR = ramus flexure right; NC = nuchal crest; MP = mastoid process; SM = supraorbital margin; GL = glabella; ME = mental eminence; MT = mental tubercles; Mprot = mental protuberance; ZE = zygomatic extension

We found the highest consistency and availability (score 2) for the traits glabella and supraorbital margin throughout all modality comparisons (Fig. 7). The performance of the other ordinal traits (nuchal crest, mastoid process, mental eminence, mental tubercles, mental protuberance and zygomatic extension) was intermediate (scores 3 and 4). The results indicate poor consistency and availability (score 4 and 5) for the ramus flexure left and right.

## Discussion

We stress that the intention of our study was not to analyze the quality of the applied scoring protocols for their reliability to predict sex. The intention was to analyze how well observers could repeat observations of the protocols on different modalities. The importance being that virtual osteological collections become more numerous alongside the existence of their analogous counterparts [17, 21–24].

Our first research question concerned the interchangeability of dry skulls and CT images of the same bones, i.e. the type of error associated with the scoring of the same cranial traits on the two modalities. Results suggested that the two modalities were, for the majority of traits, interchangeable, although with some exceptions.

The highest agreement was for the glabella and the supraorbital margin, the poorest for the ramus flexure trait. Relating to the second research question, an interesting result was the high consistency in the scorings between the two virtual modalities (CT and 3D surface scans), especially when comparing the scorings performed on virtual versus dry bone modality. One possible explanation is the lack of a tactile sensation on both virtual modalities, as opposed to the dry bone modality. Comparing a tactile and a non-tactile modality could thus yield more divergent outcomes than comparing two non-tactile modalities with each other.

We carried out the analysis of the second research question as a pilot study on a subsample of 50 specimens as compared to 223 specimens used for the first research question. Hence, a more extensive analysis focusing on the comparison of virtual modalities with each other is desirable. While the comparison of the two virtual modalities resulted in low agreement for the ramus flexure trait, the agreement for the other traits was acceptable, especially for the nuchal crest, the supraorbital margin, the glabella, the mental tubercles and the zygomatic extension. Comparing the dry bone and the surface scan modalities with each other, we obtained agreements below the acceptable threshold, except for the nuchal crest, the supraorbital margin, the glabella and the zygomatic extension. Overall, we found a superior trait consistency and availability for the glabella and supraorbital margin, an intermediate performance for the other traits (mastoid process, mental eminence, mental protuberance, mental tubercles, nuchal crest and zygomatic extension) and a relative inferior performance of the ramus flexure.

As we did not intend to analyze the traits for their sex prediction quality, but how similar or different traits are perceived in visual-tactile versus visual-only environments, it is interesting to discuss possible reasons why some traits resulted in higher intermodality agreement than others. Before discussing this issue, however, the intra- and interobserver agreements in earlier publications about the sex estimation protocols is interesting to note as it may give an indication as to why they are consistent between modalities or why they are not. Walker's interobserver agreement of the five traits (mastoid process, mental eminence, nuchal crest, glabella and supraorbital margin) yielded overall agreement of 96%, with significant differences in the scoring process for the mastoid process [4]. In the intraobserver agreement, Walker postulated a 99.5% agreement [4]. Other studies found the highest intraobserver agreement for the glabella of 78% [49] and κ values below 0.6 for the mental eminence [57]. When Langley et al. added the zygomatic extension to the above mentioned five traits, it yielded interobserver agreement results second best after the glabella [43].

The superiority of the glabella could be owing to its nature as a discernible contour viewed from a lateral perspective. The good results for the supraorbital margin might be due to the lighting and shadows on the virtual modalities, partially compensating the absence of the tactile sensation. The mastoid process performed with a score 3 in all three comparisons. While this trait was readily available, its *κ*-values were below 0.6 in all tests. Petaros et al. (2015) reported a similarly unsuccessful analysis of the mastoid process [58], while other studies agreed on its superior performance as a sex indicator [57, 59]. With an amendment of the mastoid process involving (geo)metric measurements [58, 60], repeatability and reproducibility as well as modality consistency could possibly benefit the overall performance of this trait.

The relatively poor performance of the ramus flexure traits might have originated from a general difficulty in discerning the feature. In fact, the trait has raised controversy in the literature; while the authors of the original publication insisted on the repeatability of the ramus flexure trait [42, 61], they did not test its reproducibility. Other groups attempting to reproduce the observations did not succeed [62–70]. Our results could indicate a similar difficulty with the trait per se and subsequently with its consistency between the modalities. Hence, we can assume that a sex estimation trait with a precise description tested for intra- and interobserver agreement has a chance of being consistent across modalities. If agreements are not tested and other groups are not able to repeat observations, the quality of the trait for consistency on different modalities is questionable. However, we included the ramus flexure protocol on purpose to investigate the performance of a trait that had not been tested for reproducibility. Overall, our findings indicate that the modality is not as influential on the outcome as the description of the trait [30, 52, 71]. Thus, the question may be directed at finding suitable traits to score [72, 73] that are both accurate in predicting sex as well as applicable to the analogous and the virtual environment. Our study supplies information on the latter question. Further research on the former question could now follow.

The skull is a rather robust skeletal structure, contrasting with ribs, which fracture rather easily. Hence, cranial features were generally observable in 80% to 100% of our specimens. In contrast, the often-fragmented mandibular ramus allowed observations of the ramus flexure trait in approximately a quarter of specimens only. Combined with the poor consistency of this trait between the modalities, the ramus flexure trait might not be worth investigating further.

The intermediate results for the third research question involving the mental eminence corroborated the finding of a previous study, which investigated the consistency of this trait on dry bone and micro-XCT reconstructions of 105 South African individuals from the Pretoria Bone Collection with four observers [27]. Results suggested that the mental eminence was not scored consistently on the analogous (dry bone) and the virtual (micro-XCT) modalities [27]. While a strong expression of the mental tubercles is closely linked to a square, male chin, less pronounced tubercles hint at a more rounded and female chin [44, 74–76]. Hence, since it is generally acknowledged that the menton exhibits quantifiable sexual dimorphism [76–78], this relative inconsistency between the modalities may be caused by an imprecise trait description of the mental eminence. Earlier descriptions of the mental eminence were unclear as to the exact location [3, 4], and later it was stated that *"the mental eminence is also known as the mental protuberance"* [5]. The different features constituting the menton shape, e.g. protuberance, tubercles, fossa mentalis and incurvatio mandibularis [79], may be expressed in different degrees, independent from each other. Given this intricate anatomy of the menton [49], a precise description of the trait is indispensable in order to promote its consistent scoring across modalities. At the same time, an imprecise trait definition may also lead to an unreliable sex estimation accuracy [49, 57]. This consideration, in conjunction with earlier results [27] suggested the separation of the mental eminence into two components. This led to a higher agreement for the mental tubercles as compared to the mental eminence and the mental protuberance, encouraging an investigation of that trait concerning the accuracy in predicting sex.

The recent paper investigating the modality interchangeability of sex estimation traits on the human pelvis [30] found the greatest consistency in one nonmetric and six metric traits. The iliac tuberosity [80], together with the greater sciatic notch height (adapted definition), the ischium post-acetabular length, the spino-sciatic length, the spino-auricular length, the cotylo-sciatic breadth and the vertical acetabular diameter [81] had resulted in superior consistency and availability [30]. These traits, combined with the glabella and the supraorbital margin could be merged into a new set of sex estimation traits to be tested for its sex prediction accuracy. If the traits yield satisfactory accuracies, they could be combined into a new set of traits for which the modality interchangeability has already been tested. They could then be confidently used on both the dry bone and the CT modality. Likewise, the group of pelvic (postauricular surface, postauricular space, sciatic notch, composite arch, ischio-pubic proportion, subpubic concavity, acetabulo-symphyseal pubic length, cotylo-pubic width, innominate length and iliac breadth) and cranial (nuchal crest, mastoid process, mental eminence, mental tubercles, mental protuberance and zygomatic extension) traits resulting in intermediate performance could be combined and tested for accuracies in a future study. Moreover, the consistency of age-at-death estimation traits could be another field for investigation in a future study.

Limiting factors of our study were the number of virtual modalities included and the state of bone preservation. Both observers had previous experience with the virtual modalities. Comparisons with a study including an observer without any prior experience with virtual images of bones would be interesting as levels of confidence might vary [82]. Moreover, we used different sample sizes for the analysis of our research questions. While our main focus lay on the dry bone-CT comparison, we consider the addition of 3D surface scans a pilot study, encouraging a more extensive analysis with a larger sample of 3D surface scans.

To the best of our knowledge, no research has been published so far comparing the performance of cranial traits on the analogous and the virtual modalities, encompassing a large sample.

## Conclusion

The majority of the investigated traits yielded an acceptable performance across all modalities (dry bone, CT and surface scans). We found a superior performance of the two virtual modalities when compared to each other, as opposed to the dry bone environment. However, the dry bone modality still performed within the acceptable threshold, except for the comparison with CT scans on the ordinally scored traits. Thus, we can partly confirm the modality interchangeability as investigated in this work. The degree of detail in the trait definition plays a bigger role in terms of observational error than the specific observation modality (physical versus virtual). This is likely to influence also the accuracy of the deriving sex estimates.

A combination of consistent traits from the pelvis [30] and the skull, as well as potentially other skeletal elements, should be tested for sex estimation accuracy. Consequently, a new sex estimation method could be proposed including traits that are accurate as well as consistent between the analogous and the virtual modalities.

## Data Availability

Not applicable.
